# Supplementary material for: Self-consistent solution for the magnetic exchange interaction mediated by a superconductor
Source: Sci Rep. 2021 Mar 3;11:5028. doi: 10.1038/s41598-021-83620-3 (PMC7930259; doi:10.1038/s41598-021-83620-3)
Supplement: Supplementary file 1 — Supplementary information. [file 41598_2021_83620_MOESM1_ESM.pdf]

Supplementary Information: Self-consistent solution for the magnetic exchange interaction mediated by a superconductor

Atousa Ghanbari Birgani[1], Vetle K. Risinggård[1], and Jacob Linder[1,*]

[1]Center for Quantum Spintronics, Department of Physics, Norwegian University of Science and Technology, NO-7491 Trondheim, Norway
[*]jacob.linder@ntnu.no

ABSTRACT

We here consider the effect of a self-consistently obtained exchange field on the RKKY interaction.

Selfconsistent magnetization

The following exchange Hamiltonian describes the magnetization model inside the ferromagnets:

$$H_M = \sum_{\langle i,j \rangle} J_{ij} \boldsymbol{S}_i \cdot \boldsymbol{S}_j \tag{1}$$

Here, $\boldsymbol{S}_i$ is the spin operator of the itinerant electrons and is equal to $\boldsymbol{S}_i = \sum_{\alpha\beta} \boldsymbol{\sigma}_{\alpha\beta} c^\dagger_{i\alpha} c_{i\beta}$. We can treat this nearest-neighbour exchange term with a mean-field approximation. As a result,

$$H_M = \sum_{\langle i,j \rangle} J_{ij} (\langle \boldsymbol{M}_i \rangle \boldsymbol{S}_j + \langle \boldsymbol{M}_j \rangle \boldsymbol{S}_i - \langle \boldsymbol{M}_i \rangle \langle \boldsymbol{M}_j \rangle) \tag{2}$$

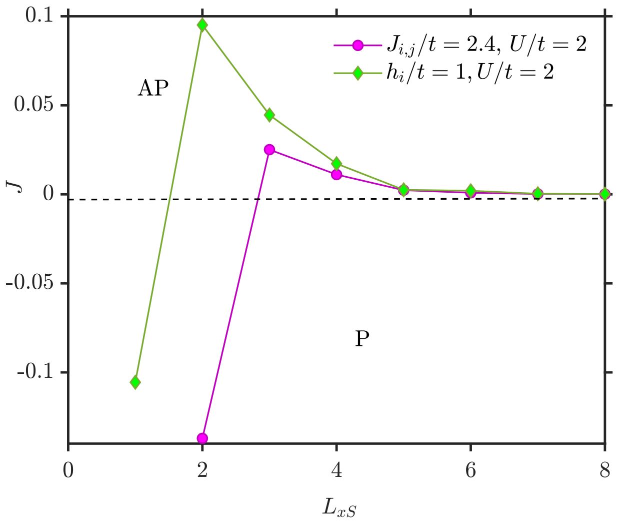

Figure 1. J vs the length of superconducting part (L_{xS}) for the F-S-F structure. Green solid line is for the case of simplified magnetization model when $L_y = 10, L_{xF} = 2, \mu_S = 0.8t, \mu_F = 0.9t, k_B T = 0.01t, |h_i^L| = |h_i^R| = h_i = 1t$. The purple solid line is when we treat magnetization model self-consistently and $L_y = 10, L_{xF} = 2, \mu_S = 0.8t, \mu_F = 0.8t, k_B T = 0.001t, J_{ij} = 2.4t$ inside the ferromagnets and at the interfaces, also $J_{ij} = 0$ inside the superconductor.

is the mean-field magnetization Hamiltonian. In this model, there will be a threshold value for J_{ij} that gives a finite value for the self-consistently solved magnetization. Therefore, it only gives us the opportunity of studying large exchange parameter values J_{ij}. Therefore, we have simplified this model in our manuscript in order to be able to consider wider range of exchange field strengths. In Phys. Rev. B **52**, 411 (1995) typical values for the Heisenberg coupling energy per junction cross-sectional area in the case of metallic spacers were found to be ~ 10 nJ/cm^2.

To compare the self-consistent solution of the mean-field Hamiltonian above to the model with fixed exchange field h used in the manuscript, we show in Fig. 1 the RKKY interaction energy for these two models. As seen, the parameters can be chosen so that the behavior is qualitatively very similar and matches even quantitatively as long as the superconductor is not very thin.
